# Foreword: Synthesis of the Greenland Ecosystem Monitoring program

**DOI:** 10.1007/s13280-016-0860-z

**Published:** 2017-01-23

**Authors:** Torben R. Christensen, Elmer Topp-Jørgensen, Mikael K. Sejr, Niels Martin Schmidt

**Affiliations:** 1 Department of Earth and Ecosystem Science, Lund University, Sölvegatan 12, 22362 Lund, Sweden; 2 Department of Bioscience, Aarhus University, Frederiksborgvej 399, 4000 Roskilde, Denmark; 3 Department of Bioscience, Aarhus University, Vejlsøvej 25, Building A2.11, 8600 Silkeborg, Denmark; 4 Arctic Research Centre, Aarhus University, Ny Munkegade, bldg 1540, 8000 Aarhus, Denmark

The Greenland Ecosystem Monitoring (GEM) program has over the past two decades established itself firmly as an internationally leading environmental barometer measuring climate change impacts and ecosystem changes in the Arctic. The program was established in 1995 at Zackenberg Research Station in a High-Arctic ecosystem. Over the past decade, GEM expanded to include a Low-Arctic site, Nuuk/Kobbefjord area, and more recently initiated the inclusion of Disko/Qeqertarsuaq at the transition between the Low-Arctic and High-Arctic.

GEM is the longest running operational ecosystem/climate-oriented monitoring program in the Arctic contributing to a deeper understanding of ecosystem change and function. The mission of GEM is threefold and embraces the following goals:

1. To contribute to a coherent and science-based description of the state of the environment, including its biodiversity, in Greenland and the Arctic in relation to climatic changes with focus on ecosystem responses and on global impacts related to feedback processes.
2. To provide science-based input on the state of the environment in Greenland and the Arctic for Danish, Greenlandic and international policy development, adaptation, and administration.
3. To provide a platform for cutting-edge inter-disciplinary research on the structure and function of arctic ecosystems.

To achieve this, the GEM program is composed of five integrated sub-programs (Climate, Geo, Bio, Marine, and Glacio) that conduct comprehensive studies of climate change and ecosystem dynamics within the domain covered by GEM (Fig. 1). The sub-programs combine long-term monitoring and short-term research projects to fully understand ecosystem dynamics and processes in a changing Arctic. To operate extensive research and monitoring operations in often remote and harsh arctic environments, close cooperation with logistics operators are needed. Integration of monitoring, research, and logistics is therefore fundamental to the success of GEM.

**Fig. 1:**
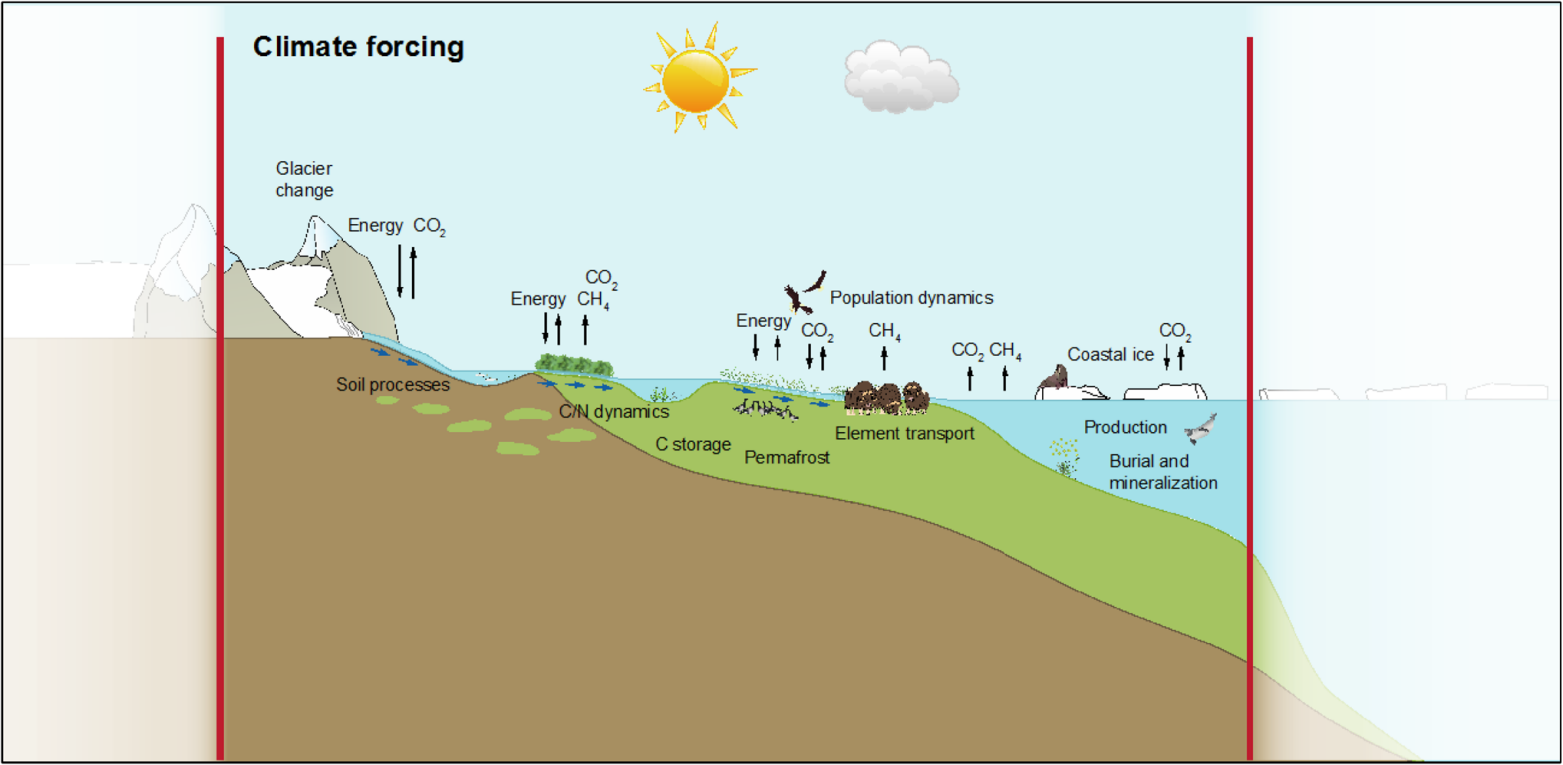
The GEM realm: studying ecosystem patterns, dynamics, and linkages in a changing climate from the coastal zone to glaciated environments on the fringes of the Greenlandic ice cap (from Christensen and Topp-Jørgensen 2016)

The program includes a multitude of climate and ecosystem variables being measured on a continuous basis. While these long time series are fundamental to GEM, some degree of flexibility is also needed to continuously implement new scientific developments (e.g., standards, methodologies or technologies) and to address potential changes in science agendas and policy needs. This flexibility is secured through an adaptive monitoring approach with annual reviews of sub-programs.

GEM is represented in numerous scientific networks, programs, and organizations to continuously influence and implement international protocols and standards.^1^ The data generated by GEM also provide input to a variety of thematic, national, arctic, and international assessments, including Arctic Councils monitoring and assessments programs (e.g. AMAP and CAFF) and international agreements (e.g. IPCC and CBD). All data generated within GEM are made freely available.^2^

While GEM historically has focused on detailed studies of a few locations, it holds significant potential for using the long-term monitoring data and process understanding for upscaling to Greenlandic scale and for strengthening applied science components through monitoring essential ecosystem components and address cumulative impacts of climate change and societal development (see Postscript for how GEM seek to achieve this; Christensen et al. 2017). With its publicly available data and leading role in monitoring arctic ecosystems, GEM is an important contributor to the assessment of the status and trends of ecosystems and the organisms therein and for understanding ecosystem processes of relevance for providing government advice on climate impacts, sustainability, and adaptation.

This special issue presents a collection of studies that attempts to synthesize GEM data across sites and disciplines. It also contains a comparison of the GEM monitoring sites to other arctic areas and thus put the program in a wider geographical context.

We trust the collected papers in this special issue will be valuable reading for anyone interested in arctic science in general and those sharing a concern and specific interest in the ecosystem impacts of climate change in particular.

## Footnotes

1
www.g-e-m.dk.

2
http://data.g-e-m.dk/.
